# Topics of Public Interest

**Published:** 1908-07

**Authors:** 


### TOPICS OF PUBLIC INTEREST.

### Buffalo to Fight Tuberculosis

### Free Open Air Camp for Consumptives

TWENTY men of Buffalo have guaranteed enough money to maintain a free open-air camp for consumptives for five months beginning on July 1st. The camp will be located on the outskirts of the city and will accommodate about 30 tuberculosis patients. It is expected to be the neucleus of a municipal tuberculosis hospital in which all the charities of the city may unite next year.

Dr. John H. Pryor, former superintendent of the state hospital for incipient tuberculosis at Raybrook, will supervise the camp, which will be in direct charge of Dr. George J. Eckel. Dr. Eckel has just made a visit to tuberculosis camps in some of the large cities and has gathered ideas.

The camp patients will be selected at the Fitch Dispensary every afternoon from 4 to 5 o'clock. Those who have no suitable accommodations to live in the open air in their homes will have preference. When a patient is accepted his family will be examined, and any members who show signs of the white plague in its incipient stages will be hustled to Raybrook.

Each morning for five months, beginning on July 1, the patients will be taken to the camp on a trolley car. They will be fed three meals a day there and will live in the open air. A building will give shelter in case of storm. Any patients seriously ill can stay over night in charge of a nurse. In this way it is hoped to start a fight against the white plague, which carries off its hundreds in Buffalo alone every year and 50,000 in New York State. By demonstrating the open-air treatment and detecting exposed cases while they are in the incipient stages, the most good can be accomplished.

Robert K. Root is president of the camp and a member of the executive committee, which includes A. D. Watson, Chauncey Hamlin, Thomas B. Lockwood and Irving P. Underhill. Mr. Underhill is secretary and treasurer of the organisation and any contributions to the fund should be sent to him in the Prudential building.

Just now the camp wants some equipment. Reclining chairs, steamer chairs, blankets and tents are in demand, and anyone who can donate any such to the work should send to the Fitch building at Swan and Michigan streets. It is estimated it will cost about $5,000 to run the camp this summer. The work of raising the money was started a month or more ago and it was announced at a meeting of the finance committee held recently that all the money has been guaranteed. The committee on site is now considering several locations which have been offered. When the camp is organised visitors will be received at any time to see the demonstration of the open-air treatment. The physicians say there is never danger of infection when the cases are handled properly.

These men are on the finance committee and have made the work possible: Robert K. Root, Howard A. Forman, William A. Douglas, Joseph P. Dudley, Chauncey J. Hamlin, Arnold Watson, Harry D. Williams, Richard Lyman, H. M. Gerrans, Eugene C. Roberts, Hugh Kennedy, Thomas B. Lockwood, Irving Underhill, William H. Fitzpatrick, Alderman John P. Sullivan, Dudley Irwin, Ralph Plumb, Harry D. Kirkover, A. Conger Goodyear, E. H. Webster, Henry Vom Berge, Hans Schmidt, Walter P. Cooke, Carl C. Machemer, John B. Williams, Robert W. Chapin, Charles B. Sears, George A. Lewis, E. L. Koons and W. Caryl Ely.

---

### Representation of the Profession in Legislative Bodies

[*Cleveland Medical Journal*, June, 1908]

THE candidacy of Charles A. L. Reed, of Cincinnati, for a seat in the United States Senate should appeal to every one who believes that the medical profession should have representation in our legislative bodies so that the necessity for legislation dealing with hygienic measures affecting the health of the whole nation can be properly and intelligently emphasised. At present Ohio is misrepresented in the United States Senate by two men whose records are of the most unstatesmanlike character. It is in the place of one of these, Senator Foraker, whose term expires next March, that the friends of Dr. Reed hope to see him given an opportunity to exercise his wide experience of public affairs. Between these two men there should be no doubt in the minds of physicians as to which would be more preferable. Senator Foraker's attitude towards the profession and toward all that it stands for is too well known to require explanation.

The qualifications of Dr. Reed for such a position and the value that his services would prove to the public at large are shown by what he has already accomplished. The passage of the Pure Food and Drug Bill was in no small degree due to his personal efforts when, as chairman of the Committee on Medical Legislation of the American Medical Association, he appeared before a committee of Congress to urge the adoption of this all-important measure. He has rendered valuable public service on numerous other occasions, such for instance as representing the United States at several International Congresses. His reports upon conditions in Panama when he acted as Special Commissioner to President Roosevelt created widespread interest and led to many important reforms.

The presence of such a representative in the United States Senate would be of the utmost value in furthering the enactment of much-needed legislation, such for example as the organisation of a National Department of Public Health. The average politician utterly fails to understand the necessity for such measures and the personnel of the United States Senate would be vastly improved by the infusion of a little new blood of a variety somewhat different from that pertaining to those members who, although eminently fitted to direct legislation favorable to certain interests they represent, do not seem to realise the value of hygienic measures directed to the public good or oppose legislation of this sort for such selfish reasons as they exhibited in opposing the Pure Food and Drug Bill and the Child Labor Bill.

It is a great pity that the election of United States Senators in this State is not directly in the hands of the people. If such were the case party lines could be much more easily ignored, and the election of such a man as Dr. Reed, as compared with either of our present Senators, rendered far more likely than it is under the present system. The question is not one of party politics at all, else we should not interfere, but it is one that appeals to all fair minded persons interested in such a legislative program as has been approved by the American Medical Association and the Ohio State Medical Association.

---

Relation of Tuberculous Cows to Tuberculosis in Children.—William Leland Stowell of New York, describes the results of the feeding of the children of some of the wards of the City Hospital for Children on Ward's Island. (*Medical Record*), on milk from a city herd that was afterward ascertained to be tuberculous. The herd was tested and all the animals in it had to be killed as the test showed some degree of tuberculous infection in all. All the children who had been fed on this milk were tested by the ophthalmo-reaction, seventy-seven in all. Of these nineteen reacted; thirteen were surgical cases of tuberculosis; and three were not suspected of tuberculosis. The author concludes that fresh, clean milk is more wholesome than pasteurised milk. The danger of infection from tuberculous milk is very slight. Less than ten per cent. mortality in the whole hospital was due to tuberculosis.

---

In persons who have had malaria any operation which taxes the vital powers may provoke a recurrence, and it is important to differentiate the resulting fever from that due to a septic process.—*International Jour. Surgery.*

